# Bright Bacterium for Hypoxia‐Tolerant Photodynamic Therapy Against Orthotopic Colon Tumors by an Interventional Method

**DOI:** 10.1002/advs.202004769

**Published:** 2021-06-18

**Authors:** Daoming Zhu, Jing Zhang, Guanghong Luo, Yanhong Duo, Ben Zhong Tang

**Affiliations:** ^1^ Department of Radiation Oncology the Second Clinical Medical College of Jinan University 1st Affiliated Hospital of Southern University of Science and Technology Shenzhen People's Hospital Shenzhen 518020 China; ^2^ Hong Kong Branch of Chinese National Engineering Research Center for Tissue Restoration and Reconstruction and Institute for Advanced Study The Hong Kong University of Science and Technology Clear Water Bay, Kowloon Hong Kong 999077 China; ^3^ Department of Laboratory Medicine Nanfang Hospital Southern Medical University Guangzhou 510515 China; ^4^ Department of Microbiology Tumor and Cell Biology (MTC) Karolinska Institutet Stockholm Sweden

**Keywords:** aggregation‐induced emission, bright bacteria, hypoxia‐tolerant photodynamic therapy, interventional photodynamic therapy, orthotopic colon tumors

## Abstract

While promising, the efficacy of aggregation‐induced emission (AIE)‐based photodynamic therapy (PDT) is limited by several factors including limited depth of laser penetration and intratumoral hypoxia. In the present study, a novel bacteria‐based AIEgen (TBP‐2) hybrid system (AE) is developed, that is able to facilitate the hypoxia‐tolerant PDT treatment of orthotopic colon tumors via an interventional method. For this approach, an interventional device is initially designed, composed of an optical fiber and an endoscope, allowing for clear visualization of the position of the orthotopic tumor within the abdominal cavity. It is then possible to conduct successful PDT treatment of this hypoxic tumor via laser irradiation, as the TBP‐2 is able to generate hydroxyl radicals (•OH) via a type I mechanism within this hypoxic microenvironment. Moreover, this interventional approach is proved to significantly impair orthotopic colon cancer growth and overcame PDT defects. This study is the first report involving such an interventional PDT strategy to knowledge, and it has the potential to complement other treatment modalities while also highlighting novel approaches to the design of hybrid AIEgen systems.

## Graphical abstract

A novel bacteria‐based AIEgen hybrid system that is able to facilitate the hypoxia‐tolerant PDT treatment of orthotopic colon tumors via an interventional method is developed. This interventional approach is proved to significantly impair orthotopic colon cancer growth and overcame PDT defects.

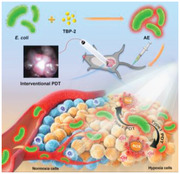

## 1 Introduction

Aggregation‐induced emission (AIE)‐active fluorophores exhibit significantly enhanced emissivity when aggregated as compared to when present as monomers.^[^ ^1^ ^]^ Owing to their high biocompatibility, high photostability, and compatibility with high‐contrast imaging strategies, AIE luminogens (AIEgens) represent an ideal bioimaging tool.^[^ ^2^ ^]^ AIEgens have also been leveraged for noninvasive cancer treatment through photodynamic therapy (PDT) approaches, as they can serve as a source of reactive oxygen species (ROS) production in response to light.^[^ ^3^ ^]^ While weekly emissive in a monomeric form, these AIEgens exhibit intensive illumination upon aggregation due to the restriction of intramolecular motion and associated radiative decay.^[^ ^4^ ^]^ The effective suppression of nonradiative AIEgens following aggregation can facilitate bioimaging, and can also improve triplet exciton stability, thereby driving robust ROS generation.^[^ ^2^, ^5^ ^]^ Several different approaches to leveraging AIEgens for targeted tumor PDT have been developed to date.^[^ ^5^, ^6^ ^]^ However, tumor treatment using AIEgens is still limited by a number of factors. For one, the laser penetration depth is limited, precluding the use of PDT to treat deep tumors and affecting tissues such as the liver or the colon.^[^ ^6^, ^7^ ^]^ In addition, hypoxia within the tumor microenvironment can limit the efficacy of PDT, and few studies have demonstrated the successful PDT treatment of hypoxic tumor tissues.^[^ ^8^ ^]^ As such, there is a clear need for the development of novel AIEgens‐based platforms capable of more reliably treating a wider range of tumor types.

Several species of bacteria including *Salmonella*, *Bifidobacterium*, and *Escherichia coli* exhibit a selective tropism for hypoxic sites that enables them to accumulate and replicate within tumor tissues.^[^ ^9^ ^]^ Owing to the high selectivity of these bacteria for tumor sites, they can be reliably leveraged for the precise targeting of hypoxic tumor tissues.^[^ ^10^ ^]^ We have previously reported the development of an AIE photosensitizer (PS) with two positive charges (termed TBP‐2) that is able to selectively target and bind to *E. coli* via uptake into the periplasmic space within these bacteria.^[^ ^2a^ ^]^ Since *E. coli* also exhibits a hypoxic tumor tissue tropism according to other research,^[^ ^9^, ^10^, ^11^ ^]^ we therefore sought to leverage TBP‐2‐loaded *E. coli* for the treatment of hypoxic tumors. AIEgen‐loaded bacterial enrichment offers promise as a means of selectively targeting hypoxic tumors. However, the hypoxic intratumoral microenvironment limits the anticancer efficacy of PDT.^[^ ^12^ ^]^ In general, PDT relies upon the transformation of ground triplet‐state molecular oxygen (^3^O_2_) into reactive singlet oxygen (^1^O_2_) via a type II mechanism that is highly dependent upon local O_2_ concentrations.^[^ ^13^ ^]^ Low O_2_ levels within solid tumors can thus compromise PDT efficacy, particularly in patients requiring sustained or prolonged treatment.^[^ ^14^ ^]^ In contrast, however, hypoxia‐tolerant type I PDT mechanisms can overcome these limitations by markedly lowering O_2_ requirements, as O_2_ is partially recycled during these cascade reactions, thereby ameliorating intratumoral hypoxia and enhancing PDT efficacy.^[^ ^15^ ^]^ We found that TBP‐2 was able to mediate PDT effectively via both type I and type II mechanisms through the generation of multiple forms of ROS including hydroxyl radicals (•OH) and singlet oxygen (^1^O_2_) in response to light irradiation in this experiment. TBP‐2‐loaded bacteria (AE) can thus not only effectively traffic to hypoxic tumors, but also represent an ideal tool for conducting PDT‐mediated cancer treatment via a type I mechanism.

PDT is additionally limited by the intrinsically poor ability of light to penetrate into tissues, rendering this approach ineffective for the management of tumors within deep‐seated tissues such as the liver or the colon.^[^ ^8^, ^16^ ^]^ We thus hypothesized that a locoregional interventional approach to PDT may represent a more clinically viable means of treating abdominal tumors. To date, no studies have compared the efficacy of such an interventional PDT regimen with other therapeutic modalities, underscoring the importance of conducting such a study.

**Scheme 1:**
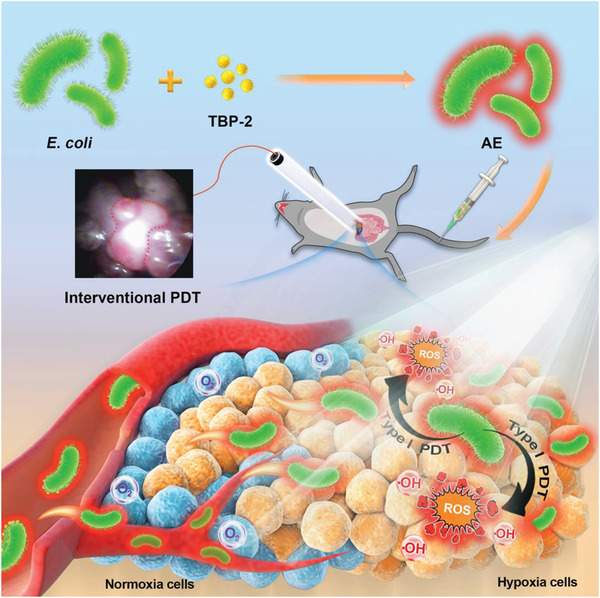
Schematic illustration of bright bacteria for hypoxia‐tolerant photodynamic therapy against orthotopic colon tumors by an interventional method.

In the present study, we thus developed a novel bacteria‐based hybrid AIEgen system (AE) capable of mediating the hypoxia‐tolerant PDT treatment of orthotopic colon tumors via an interventional method (**Scheme** **1**). We began by developing a novel interventional device composed of an endoscope and an optical fiber that was able to reliably visualize the orthotopic tumor within the abdominal cavity. Hypoxia‐targeting *E. coli* were then leveraged to deliver TBP‐2 into the hypoxic core regions of these tumors, thus enabling the effective intratumoral accumulation of this AIEgen. PDT was then successfully conducted via laser irradiation using our interventional system, with TBP‐2 serving as a PS to facilitate •OH generation via a type I mechanism within this severely hypoxic microenvironment. This approach was sufficient to markedly inhibit orthotopic colon tumor growth and to overcome many of the traditional limitations of PDT. In summary, this novel approach to the interventional treatment of deep tumors offers promise as a complement to other therapeutic modalities, and may additionally serve as a foundation for the development of further novel hybrid AIEgen systems.

## 2 Results and Discussion

**Figure 1:**
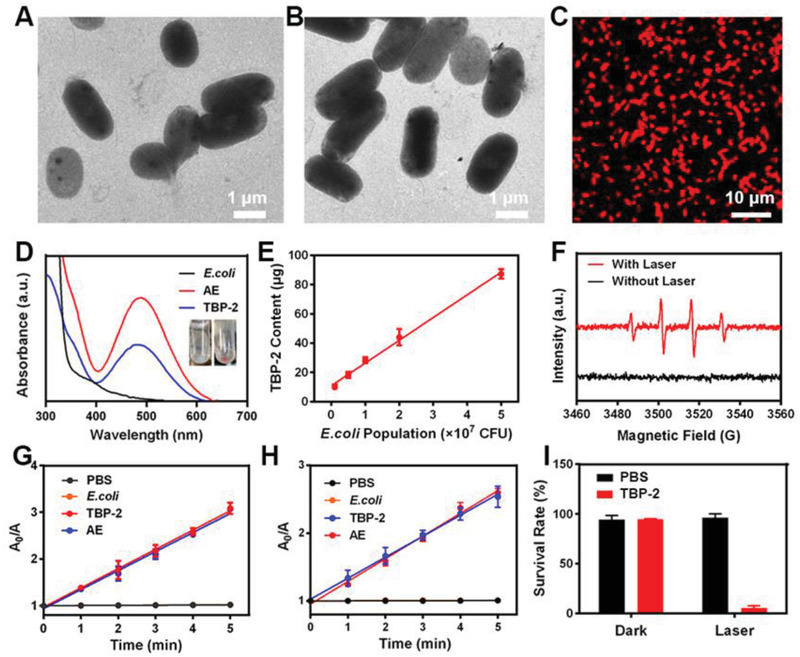
TEM images of A) *E.coli* and B) AE. C) Fluorescent images of AE, indicating successful TBP‐2 loading into these bacteria. *λ*_ex_ = 488 nm and *λ*_em_ = 600–700 nm. D) Absorption spectra of *E.coli* (in PBS), AE (in PBS), and TBP‐2 (1% DMSO fraction). E) TBP‐2 levels in different *E.coli* populations. F) TBP‐2‐mediated •OH generation with/without white laser (WL, 0.1 W cm^−2^, 5 min) excitation via ESR. G) 9,10‐Anthracenediyl‐bis(methylene)‐dimalonic acid (ABDA) decomposition rates induced by ^1^O_2_ generation associated with the indicated formulations under WL excitation (0.1W cm^−2^, 5 min, TBP‐2 was dissolved in 1% DMSO solution). H) TA decomposition rates induced by •OH generation associated with the indicated formulations under WL excitation (0.1W cm^−2^, 5 min, TBP‐2 was dissolved in 1% DMSO solution). I) Assessment of the antibacterial effect of TBP‐2 on *E. coli* under different lighting conditions.

We began by successfully preparing and characterizing TBP‐2 (Figure S1, Supporting Information).^[^ ^2a^ ^]^ We then combined TBP‐2 with *E. coli* and found that it was readily absorbed by these bacteria within 30 min owing to its two positive charges, which can readily interact with the negatively charged LPS on the bacterial surface to replace the divalent lipid cations. This destabilizes the LPS coating and causes partial permeabilization of the bacterial surface such that TBP‐2 can penetrate into the periplasmic space. Importantly, the morphology of *E. coli* was comparable before and after TBP‐2 absorption (**Figure** **1**A,B), and AE preparations exhibited bright red diffuse fluorescence following 488 nm laser irradiation (Figure 1C). After loading TBP‐2, the zeta potential of AE was greater than that of pure *E.coli* (Figure S2, Supporting Information). Absorption spectrum analyses revealed that AE exhibited an identical characteristic absorption peak to that of TBP‐2 at 493 nm (Figure 1D). Together, these data revealed that TBP‐2 was readily absorbed by bacteria, thereby facilitating successful AE preparation. TBP‐2 levels also rose with increasing bacterial concentrations at an initial TBP‐2 content of 100 µg (Figure 1E). To confirm the utility of TBP‐2 as a type I PS, we utilized an electron spin resonance (ESR) test and confirmed that TBP‐2 efficiently mediated hydroxyl radical production in response to white light laser (WL) exposure. The results show that TBP‐2 can produce •OH efficiently under light, which is the most direct evidence of TBP‐2 as a type I PS. 9,10‐Anthracenediyl‐bis(methylene)‐dimalonic acid (ABDA) and terephthalic acid (TA) degradation experiments additionally confirmed the ability of AE to efficiently generate singlet oxygen and hydroxyl radicals, in line with prior results (Figure 1G,H). TA was used to track the capture of •OH and generate 2‐hydroxy TA, along with emitting 435 nm fluorescence light.^[^ ^17^ ^]^ It should be noted that AE did not affect the singlet oxygen and hydroxyl radical production capacity of TBP‐2. We further confirmed the phototoxic effect of TBP‐2 on *E. coli* in response to light exposure (Figure 1I). While *E. coli* were unaffected by this AIEgen in the absence of light, the survival of these bacteria was markedly compromised upon light exposure, consistent with the ability of TBP‐2 to produce high levels of ROS and to thereby induce phototoxicity.^[^ ^2a^ ^]^ Bacterial structures can also be directly destroyed upon light irradiation (Figure S3, Supporting Information), thereby enhancing TBP‐2 release (Figure S4, Supporting Information). After a long time of culture or many times of washing, TBP‐2 can still remain on the AE membrane, indicating that AE has good stability (Figures S5 and S6, Supporting Information). In summary, these data indicated that we were able to successfully generate an AE preparation capable of simultaneously producing singlet oxygen and hydroxyl radicals in response to light. We thus leveraged this AE preparation in order to explore its antitumor utility. In general, AE was prepared by a very simple and environmental‐friendly method, from which we thought that almost all materials with affinity to bacteria could be used for tumor treatment in this way. So this idea is very meaningful for the future tumor treatment system.

**Figure 2:**
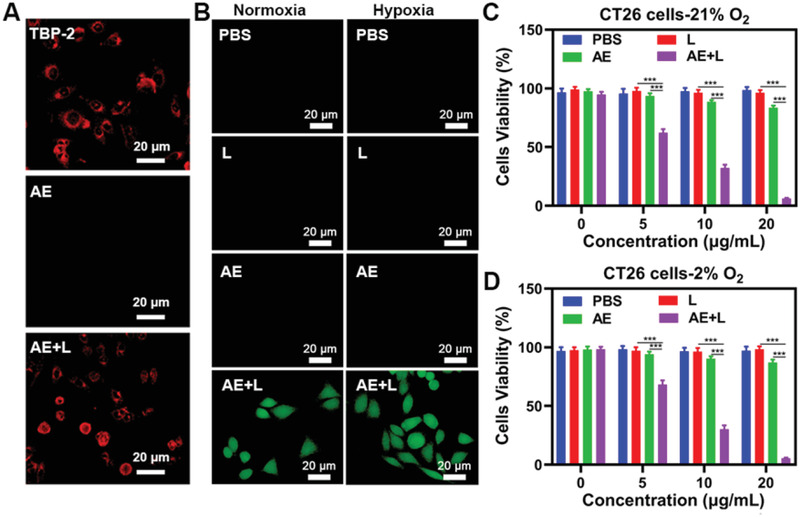
A) CT26 cell fluorescence images following the indicated treatments. *λ*_ex_ = 488 nm and *λ*_em_ = 600–700 nm. B) Tumor cells DCFH‐DA fluorescence images were observed after the indicated treatments under normoxia or hypoxia condition. L: white light (0.1 W cm^−2^, 10 min). C) Under normoxia or D) hypoxic conditions, viability of CT26 tumor cells treated with PBS, L (0.1 W cm^−2^, 10 min), AE, or AE+L at different TBP‐2 concentrations. *P<0.05, **P<0.01, ***P<0.001; Student's *t*‐test.

We then evaluated the in vitro antitumor ability of AE. When we assessed these interactions in vitro, we found that TBP‐2 released from AE in the presence of white light was able to rapidly interact with CT26 cancer cells (**Figure** **2**A). We used cell membrane dye (DiO) and TBP‐2 co‐localization found that TBP‐2 mainly existed on the cell membrane (Figure S7, Supporting Information). This is in line with prior studies indicating that molecules bearing two positive charges can more readily adhere to tumor cell membranes.^[^ ^18^ ^]^ However, AE treatment alone was insufficient to mediate TBP‐2 binding to tumor cell membranes,^[^ ^18^ ^]^ which was only evident following light exposure. This suggests that TBP‐2 is initially bound very tightly to bacteria such that it can only interact with surrounding tumor tissues following light‐mediated release from AE. For determination of ROS levels via fluorescent imaging, CT26 cells were incubated for 0.5 h with 4 different groups: 1) PBS; 2) WL (L, 0.1 w cm^−2^, 10 min); 3) AE; 4) AE+L. The TBP‐2 concentration was 10 µg mL^−1^ in groups 3 and 4. We found that under both hypoxic and normoxic conditions, AE was able to produce large volumes of ROS within tumor cells upon light exposure in vitro, consistent with the ability of AE to facilitate PDT in an oxygen‐independent manner attributable to effective TBP‐2‐mediated hydroxyl radical production (Figure 2B). No obvious singlet oxygen sensor green (SOSG) signal associated with ^1^O_2_ was observed in hypoxic condition, whereas numerous ^1^O_2_ was generated in CT26 cells under the normoxic environment (Figure S8, Supporting Information). 3′‐(4‐hydroxyphenyl) fluorescein (HPF) was used to monitor the •OH generation, and strong fluorescence of HPF was presented in both hypoxic and normoxic conditions after light irradiation. These results indicate that AE can produce large amounts of hydroxyl radicals through type I PDT under hypoxic conditions. Importantly, AE was able to efficiently suppress tumor cell growth in a light‐ and concentration‐dependent manner (Figure 2C,D). These findings demonstrate that AE is able to efficiently mediate hypoxia‐tolerant PDT in vitro and the •OH generation are the ones that kill tumor cells under hypoxic conditions. The tumor microenvironment is hypoxic, so this conclusion is very important and provides a basis for the subsequent treatment of hypoxic tumors in vivo.

**Figure 3:**
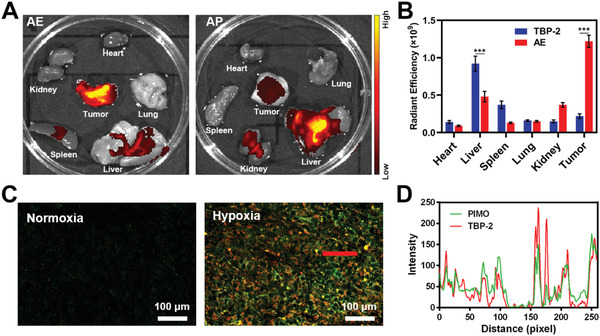
A) Ex vivo images and B) radiant efficiency in collected tumor tissues and organs in mice bearing orthotopic CT26 tumors at 24h post‐AE or AP injection. C) Fluorescent visualization of AE co‐localization with hypoxic regions. (Green: PIMO, red: TBP‐2). D) The PIMO and TBP‐2 fluorescence distribution profiles along the red line within the hypoxic region marked in Figure 3C. *P<0.05, **P<0.01, ***P<0.001; Student's *t*‐test.

We next conducted an AE biodistribution study in vivo. We began by intravenously injecting animals with TBP‐2 loaded into poly(lactic‐co‐glycolic) acid (PLGA) nanoparticles (AP), as TBP‐2 cannot be directly administered intravenously (Figures S9–S11, Supporting Information).^[^ ^2a^ ^]^ PLGA, a biodegradable synthetic polymer approved for clinical use by US FDA has been widely used in drug delivery.^[^ ^19^ ^]^ These AP particles exhibit a diameter of ≈100 nm and AP has the characteristic absorption of TBP‐2, which indicates the successful loading of TBP‐2. Following the injection of either AP or AE loaded with equivalent amounts of TBP‐2 (**Figure** **3**A,B), bright fluorescence was observed in colon tumors in animals injected with AE but not AP, consistent with the ability of AE to target tumors in vivo. Liver fluorescence in the AE group was also significantly reduced relative to that in the AP group, underscoring the problematic hepatic accumulation of traditional polymer materials. In addition, we studied the bio‐distribution in vitro at different time points after AE injection, and found that AE had a lot of accumulation in the liver at the beginning. As time went on, liver accumulation gradually decreased, while tumor accumulation increased (Figure S12, Supporting Information). This phenomenon is consistent with previous research.^[^ ^10b^ ^]^ We then conducted in vivo experiments to verify the systemic accumulation of AE after oral administration (Figure S13, Supporting Information). After 24 h of oral administration, the accumulation of AE in the tumor site was significantly less than that of intravenous administration. This is probably because it takes longer to get into the bloodstream. The specific reasons need to be further analyzed. This result also suggests that intravenous administration is more efficient in this model. Immunofluorescent analyses of tumor tissues from animals in the AE treatment group were additionally conducted, revealing that AE accumulated primarily within hypoxic tumor tissues and was largely absent within normoxic regions (Figure 3C,D). These findings were consistent with prior reports,^[^ ^9^, ^10^, ^11^, ^20^ ^]^ and underscore the ability of this AE system to target hypoxic tumors, enabling us to conduct subsequent therapeutic experiments.

**Figure 4:**
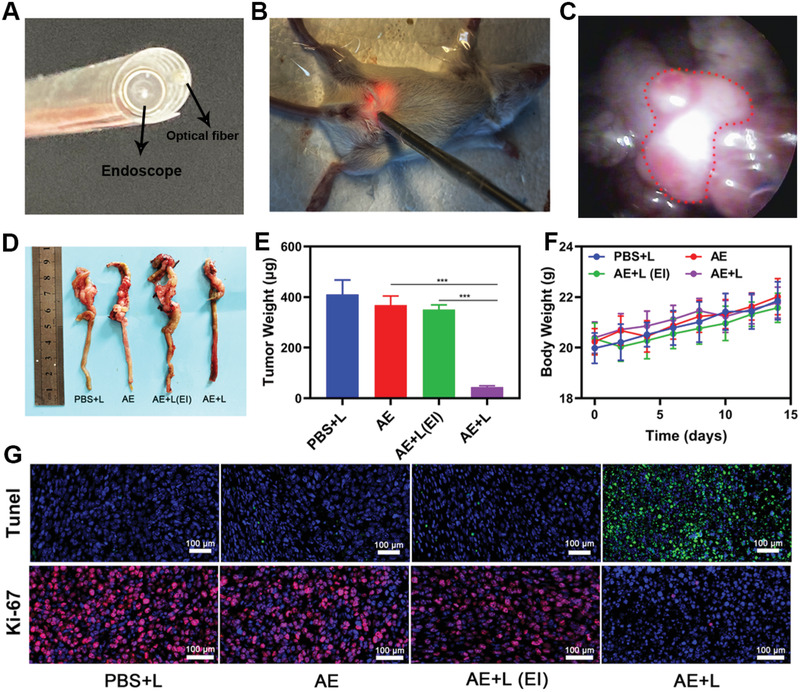
A) Development of an interventional device composed of an endoscope and an optical fiber. B) Images of mice bearing orthotopic CT26 tumors undergoing interventional PDT. C) Orthotopic tumor position within the abdominal cavity as visualized via endoscopy. The tumor is marked using a red dashed line. D) Images of resected orthotopic CT26 tumors in the indicated treatment groups. E) Tumor volume and F) body weight curves following the indicated treatments in mice bearing orthotopic CT26 tumors. G) Representative TUNEL and Ki‐67 stained tumor sections from mice in the indicated treatment groups. *P<0.05, **P<0.01, ***P<0.005; Student's *t*‐test.

Since AE showed a good targeting property for orthotopic colon cancer, we can't wait to verify its anti‐tumor ability. As we all know, orthotopic colon cancer is deep in the abdominal cavity, and the laser doesn't go that far, so we thought about using interventional light to conduct PDT. We next designed an interventional therapy device so that we could leverage these AE preparations for the treatment of orthotopic colon cancer tumors. This homemade interventional device was composed of an endoscope and a laser optical fiber joined using medical tape (**Figure** **4**A). During treatment, the abdomen was punctured with a puncture needle, which was subsequently removed. The interventional device was then inserted into the abdominal cavity along the hole made by puncture needle, and the endoscope was used to locate the tumor, after which the laser was used for tumor irradiation (Figure 4B). The endoscope allowed for clear visualization of the tumors in these mice (Figure 4C). When tumors had grown to a size similar to the diameter of the intestines, tumor‐bearing mice were divided randomly into 4 groups (each group included 5 mice): 1) PBS + interventional white laser (L); 2) AE; 3) AE + external white laser irradiation (EI); 4) AE+L. The TBP‐2 dose was 5 mg kg^−1^ in groups 2, 3, and 4. The PDT (0.1 W cm^−2^, 20 min) was performed for 24 h after intravenous injection. The treatment was conducted every 4 days for 14 days. We found that tumors in mice treated with AE and interventional laser irradiation were significantly reduced in size relative to those in the control group (Figure 4D,E), whereas therapeutic efficacy in administered AE and external laser irradiation group was negligible. This confirms that low‐power lasers are unable to penetrate the abdominal cavity, making them of limited value for the PDT treatment of deep tumors. In contrast, our interventional approach was highly efficacious. Murine body weight was largely unchanged over the course of the 14 days of treatment, consistent with a lack of treatment‐associated systemic toxicity. Similarly, no significant abnormalities were observed upon H&E staining and evaluation of heart, liver, spleen, lung, or kidney sections from these animals, and liver and kidney function were not compromised in these animals (Figures S14–S17, Supporting Information). TUNEL and H&E staining of tumor tissues revealed that tumors in the interventional treatment group exhibited greater levels of apoptotic cell than did those in control animals (Figure 4G), confirming the ability of interventional PDT to inhibit tumor growth. In summary, we have demonstrated the ability of our interventional treatment approach to successfully treat deep intraperitoneal tumors via PDT. In the treatment of deep tumors, PDT has been limited by the depth of light penetration. Our AE system provides a new idea for the photodynamic treatment of orthotopic tumors. This novel AIEgen based system has achieved potent tumor inhibition by combination with the innovative treatment device.

## 3 Conclusions

In conclusion, we herein designed a novel bacteria‐based hybrid AIEgen system capable of facilitating hypoxia‐tolerant PDT treatment of orthotopic colon tumors via an interventional method. This hypoxia‐targeted AE was utilized to specifically deliver TBP‐2 into hypoxic regions within tumors, after which laser irradiation‐mediated PDT was conducted using a novel interventional treatment system. Through this approach, the intratumoral TBP‐2 was able to serve as a PS and to thereby generate •OH via a type I mechanism under hypoxic conditions in response to laser irradiation. This approach was sufficient to inhibit orthotopic colon tumor growth and to overcome many of the limitations associated with PDT. This is also the first study to our knowledge to have employed interventional PDT to treat deep tumors, and our approach may offer value as a complement for other treatment modalities, providing novel avenues for the development of an additional hybrid AIEgen system. In the future, we will continue to design more advanced AIEgen‐based systems in order to better improve the clinical utility of these AIEgens.

## Conflict of Interest

The authors declare no conflict of interest.

## Supporting information

Supporting Information

## Data Availability

Research data are not shared.

## References

[1] a) J. Qi , Y. Fang , R. T. K. Kwok , X. Zhang , X. Hu , J. W. Y. Lam , D. Ding , B. Z. Tang , ACS Nano 2017, 11, 7177; 28692799 10.1021/acsnano.7b03062

[2] a) X. Shi , S. H. P. Sung , J. H. C. Chau , Y. Li , Z. Liu , R. T. K. Kwok , J. Liu , P. Xiao , J. Zhang , B. Liu , J. W. Y. Lam , B. Z. Tang , Small Methods 2020, 4, 2000046;

[3] a) Y. Li , Q. Wu , M. Kang , N. Song , D. Wang , B. Z. Tang , Biomaterials 2020, 232, 119749; 31918230 10.1016/j.biomaterials.2019.119749

[4] a) D. Zhu , Y. Duo , S. Meng , Y. Zhao , L. Xia , Z. Zheng , Y. Li , B. Z. Tang , Angew. Chem., Int. Ed. 2020, 59, 2; 10.1002/anie.202003672 32367646

[5] F. Gao , J. Wu , H. Gao , X. Hu , L. Liu , A. C. Midgley , Q. Liu , Z. Sun , Y. Liu , D. Ding , Y. Wang , D. Kong , X. Huang , Biomaterials 2020, 230, 119635. 31767443 10.1016/j.biomaterials.2019.119635

[6] a) D. Zhu , Y. Duo , M. Suo , Y. Zhao , L. Xia , Z. Zheng , Y. Li , B. Z. Tang , Angew. Chem., Int. Ed. Engl. 2020, 59, 13836; 32367646 10.1002/anie.202003672

[7] a) Y. Hu , C. Chi , S. Wang , L. Wang , P. Liang , F. Liu , W. Shang , W. Wang , F. Zhang , S. Li , H. Shen , X. Yu , H. Liu , J. Tian , Adv. Mater. 2017, 29, 1700448; 10.1002/adma.201700448 28682465

[8] a) W. Yu , T. Liu , M. Zhang , Z. Wang , J. Ye , C. X. Li , W. Liu , R. Li , J. Feng , X. Z. Zhang , ACS Nano 2019, 13, 1784; 30698953 10.1021/acsnano.8b07852

[9] W. Chen , Y. Wang , M. Qin , X. Zhang , Z. Zhang , X. Sun , Z. Gu , ACS Nano 2018, 12, 5995. 29786420 10.1021/acsnano.8b02235

[10] a) S. B. Wang , X. H. Liu , B. Li , J. X. Fan , J. J. Ye , H. Cheng , X. Z. Zhang , Adv. Funct. Mater. 2019, 29, 1904093;

[11] a) D.‐W. Zheng , Y. Chen , Z.‐H. Li , L. Xu , C.‐X. Li , B. Li , J.‐X. Fan , S.‐X. Cheng , X.‐Z. Zhang , Nat. Commun. 2018, 9, 1680; 29700283 10.1038/s41467-018-03233-9 PMC5920064

[12] a) S. Li , K. Gu , H. Wang , B. Xu , H. Li , X. Shi , Z. Huang , H. Liu , J. Am. Chem. Soc. 2020, 142, 5649; 32115944 10.1021/jacs.9b12929

[13] L. P. Zhao , R. R. Zheng , H. Q. Chen , L. S. Liu , X. Y. Zhao , H. H. Liu , X. Z. Qiu , X. Y. Yu , H. Cheng , S. Y. Li , Nano Lett. 2020, 20, 2062. 32096643 10.1021/acs.nanolett.0c00047

[14] C. Shi , M. Li , Z. Zhang , Q. Yao , K. Shao , F. Xu , N. Xu , H. Li , J. Fan , W. Sun , J. Du , S. Long , J. Wang , X. Peng , Biomaterials 2020, 233, 119755. 31927233 10.1016/j.biomaterials.2020.119755

[15] a) T. Luo , K. Ni , A. Culbert , G. Lan , Z. Li , X. Jiang , M. Kaufmann , W. Lin , J. Am. Chem. Soc. 2020, 142, 7334; 32248686 10.1021/jacs.0c02129

[16] a) C. Wang , P. Zhao , G. Yang , X. Chen , Y. Jiang , X. Jiang , Y. Wu , Y. Liu , W. Zhang , W. Bu , Mater. Horiz. 2020, 7, 1180;

[17] X. Pan , L. Bai , H. Wang , Q. Wu , H. Wang , S. Liu , B. Xu , X. Shi , H. Liu , Adv. Mater. 2018, 30, 1800180. 10.1002/adma.201800180 29672956

[18] D. Wang , H. Su , R. T. K. Kwok , X. Hu , H. Zou , Q. Luo , M. M. S. Lee , W. Xu , J. W. Y. Lam , B. Z. Tang , Chem. Sci. 2018, 9, 3685. 29780499 10.1039/c7sc04963c PMC5935061

[19] a) Q. Chen , L. Xu , C. Liang , C. Wang , R. Peng , Z. Liu , Nat. Commun. 2016, 7, 13193; 27767031 10.1038/ncomms13193 PMC5078754

[20] S. Xie , L. Zhao , X. Song , M. Tang , C. Mo , X. Li , J. Controlled Release 2017, 268, 390. 10.1016/j.jconrel.2017.10.041 29101053

