# Supplementary material for: Bright Bacterium for Hypoxia‐Tolerant Photodynamic Therapy Against Orthotopic Colon Tumors by an Interventional Method
Source: Adv Sci (Weinh). 2021 Jun 18;8(15):2004769. doi: 10.1002/advs.202004769 (PMC8336512; doi:10.1002/advs.202004769)
Supplement: Supplementary file 1 — Supporting Information [file ADVS-8-2004769-s001.pdf]

© 2021 Wiley-VCH GmbH

ADVANCED SCIENCE
Open Access

Supporting Information

for *Adv. Sci.*, DOI: 10.1002/advs.202004769

Bright bacteria for O_2-independent photodynamic therapy against orthotopic colon tumors by an interventional method

Daoming Zhu, Jing Zhang, Guanghong Luo, Yanhong Duo and Ben Zhong Tang**

Supporting Information

Bright bacteria for O_2-independent photodynamic therapy against orthotopic colon tumors by an interventional method

Daoming Zhu, Jing Zhang, Guanghong Luo, Yanhong Duo and Ben Zhong Tang**

Dr. D. Zhu, Dr. G. Luo and Prof. Y. Duo
Department of Radiation Oncology, the Second Clinical Medical College of Jinan University, 1st Affiliated Hospital of Southern University of Science and Technology, Shenzhen People's Hospital, Shenzhen 518020, China.
E-mail: yanhong.duo@ki.se

Dr. J. Zhang, Prof. B. Z. Tang
Hong Kong Branch of Chinese National Engineering Research Center for Tissue Restoration and Reconstruction and Institute for Advanced Study
The Hong Kong University of Science and Technology
Clear Water Bay, Kowloon, Hong Kong 999077, China.
E-mail: tangbenz@ust.hk

Dr. J. Zhang
Department of Laboratory Medicine, Nanfang Hospital, Southern Medical University, Guangzhou, 510515, China.

D. Z., J. Z. and G. L contributed equally to this work.

Materials. Phosphate buffer solution (PBS) was obtained from Thermo-Fisher (Waltham, MA, USA). 3'-(4-hydroxyphenyl) fluorescein (HPF), 9, 10-Anthracenediyl-bis (methylene) dimalonic acid (ABDA) and p-phthalic acid (TA) were obtained from Sigma-Aldrich (USA). Singlet oxygen sensor green (SOSG) and DiO (DiOC18(3)) were obtained from Dalian Meilun Biotechnology Co., LTD (China). Reactive Oxygen Species Assay Kit wsa purchased from Beyotime Company (China). LB nutrient agar was obtained from Qingdao Hope Bio-

Technology. All of the aqueous solutions were prepared using purified deionized (DI) water purified with a purification system (Direct-Q3, Millipore, USA). The other solvents used in this work were purchased from Sinopharm Chemical Reagent (China) and Aladdin-Reagent (China).

Instrument. A white light laser (consists of three different wavelengths of laser light) with an 1mm-diameter optical fiber was purchased from Ningbo Yuanming Laser Technology Co. LTD (China). A medical endoscope was purchased from Le Weishi Technology Co., LTD (China). The optical fiber and the endoscope were strapped together with medical tape. For interventional PDT, the abdominal cavity was first perforated with a 18-gauge PTC needle (Zhuhai Hokai Biomedical Electronics Co., Ltd., China), and then the endoscope and optical fiber were inserted for treatment.

Synthesis of TBP-2[1]. Into a 100 mL two-necked round bottom flask equipped with a condenser, was dissolved TBP (100 mg, 0.219 mmol) in 10 mL acetonitrile. (3-Bromopropyl) trimethylammonium bromide (85 mg, 0.329 mmol) was then added and the mixture was heated to reflux for 8 h. After cooling to room temperature, the mixture was poured into diethyl ether. The dark red precipitates formed were filtered by suction filtration to afford the desired product (125 mg, 80%). ^{1}H NMR (400 MHz, DMSO-d_6), δ (TMS, ppm): 9.28 (2H, d), 8.96 (2H, d), 8.48 (1H, d), 8.13-8.04 (3H, m), 7.38-7.36 (4H, m), 7.15-7.09 (8H, m), 4.75 (2H, m), 3.51-3.47 (2H, m), 3.13 (9H, s), 2.57-2.53 (2H, m).

Cell line and bacteria. CT26 mouse colon cancer cell line was obtained from the Cell Bank of the Chinese Academy of Sciences and incubated in RPMI-1640 medium supplemented with 10% FBS in a humidified atmosphere at 37 °C. Cell cultures under normoxic conditions (pO_2: 21%) were maintained in a humidified incubator at 37℃ in 5% CO_2 and 95% air. Hypoxic conditions (pO_2: 2%) were produced by placing cells in a hypoxic incubator (Moriguchi, Japan) in a mixture of 2% O_2, 5% CO_2, and 93% N_2. Escherichia coli Nissle 1917 (ECN) was obtained from Qilu hospital of Shandong University. *E. coli* were planted on lysogeny broth (LB) agar plates and were inoculated at 37 °C.

Establishment of orthotopic colon tumor mouse models. The Balb/c mice were anesthetized by 1% pentobarbital intraperitoneal injection. After anesthesia, the mice were fixed on sterile gauze in supine position.The hair of the operative part was removed, the skin was disinfected by iodopor, and 95% alcohol was deiodized. A longitudinal incision was made at the lower margin of the abdominal line, skin and peritoneum were cut layer by layer, and the bladder was exposed. The bladder and small intestine were removed with forceps. The large intestine segment was located deep in the abdomen. 25 μL cell suspension was extracted

with an insulin syringe. The colon was gently picked out with an ophthalmic tweezer and the CT26 cells (1×10^8/mL) was inserted parallel to the surface with a handheld syringe. It penetrates into the serosal layer of the colon and pushes the cell suspension into the submembrane. After the injection, hold for a moment, pull the needle slowly, and gently press the pinhole with sterile gauze for 10s to prevent the liquid from flowing out. Then close the abdominal cavity.

Preparation and characterization of *E.coli* / AIEgen hybrid system (AE). TBP-2 (50 μg, 10μL DMSO) was added into 2.5 mL *E. coli* suspension (1×10^7 CFU), and then placed at 37 °C with shaking (200 rpm) for 0.5 h. The AE was washed with Luria-Bertani (LB) medium and centrifuged twice (5000rpm).The morphology structures of *E. coli*, and AE were observed by the TEM (JEOL-2100). UV-vis spectra of different samples and TBP-2 content in AE were measured by the UV-vis spectrophotometry Lambda 35 (Perkin-Elmer). AE were also imaged by a confocal laser scanning microscope (CLSM, Zeiss LSM 710). Capture condition: λex = 488 nm, λem = 600–700 nm. The zeta potential of *E. coli* and AE were measured by dynamic light scattering (DLS, Nano-Zen 3600, Malvern Instruments, UK). The stability of adsorption after multiple PBS washing was verified by UV-vis spectra. The changes of AIEgen loading was also studied by UV-vis spectra in the process of bacterial proliferation in vitro.

Preparation and characterization of PLGA/AIEgen hybrid nanoparticles (AP). Briefly, TBP-2 was dissolved in DMSO at 10 mg/mL. A total of 100 μL of TBP-2 was added to 1ml PLGA (5 mg/mL) dissolved in dichloromethane. Then, the mixture was homogenized with 0.4 ml 5% w/v PVA solution for 10 min using probe sonication. The o/w emulsion was then added to 2.1 ml of a 5% w/v solution of PVA to evaporate the organic solvent for 4 h at room temperature. DPS nanoparticles were obtained after centrifugation at 3,500g for 20 min. The amount of DCPy loaded into DPS was calculated from a calibration curve acquired from UV−vis spectrophotometer measurements based on the absorbance intensity at 493 nm. The size and size distribution of DPS were measured by dynamic light scattering. The morphology structures of AP were observed by the TEM (JEOL-2100). UV-vis spectra of AP was recorded by the UV-vis spectrophotometry Lambda 35 (Perkin-Elmer).

ESR measurements. hydroxyl radical (•OH) generation was evaluated by DMPO. 10 μL DMPO was mixed with 50 μL TBP-2 at 10 μg/mL and irradiated by wight laser (WL, 0.1W/cm^2, 2 min). The signals of •OH can be shown by the ESR spectrometer. As a comparison, the TBP-2 group without laser was detected too.

Singlet oxygen ($^1O_2$)-Generation Detection. ABDA was used as the $^1O_2$-monitoring agent[2]. In the experiments, 10 μL of ABDA stock solution (7.5 mM) was added to 2 mL of TBP-2

suspension or AE or PBS or *E.coli* (equivalent 10 μg/mL TBP-2) and WL (0.1W/cm^2, 5 min) was employed as the radiation source. The absorption of ABDA at 378 nm was recorded at various irradiation times to obtain the decay rate.

•OH Generation Detection. TA was used as the •OH monitoring agent[3]. In the experiments, 300 μL of TA solution was added to 2 mL of TBP-2 suspension or AE or PBS or *E.coli* (equivalent 10 μg/mL TBP-2) and WL (0.1W/cm^2, 5 min) was employed as the radiation source. The absorption of TA at 425 nm was recorded at various irradiation times to obtain the decay rate.

TBP-2 release study The *in vitro* TBP-2 release profile of AE was carried out by dialysis method. 1 mL of AE containing 0.05 mg TBP-2 was added into 1× PBS (pH=7.4) under horizontal shaking at 100 rpm for 3 h. To investigate the stimuli effect of WL radiation on the release behavior, the release experiment of AE was initially performed with or without WL radiation (0.1W/cm^2, 5 min). At appropriate time point, the solution was centrifuged at 5000rpm for 5min, 100 μL of supernatant were collected, and an UV−vis spectrophotometer was used to monitor the released TBP-2 content. For cancer cell imaging study, CT26 cells were incubated for 0.5 h with 3 different groups: (1) TBP-2; (2) AE ; (3) AE+ white light laser (L, 0.1w/cm^2, 10min). The TBP-2 concentration was 10 μg/mL. Then, cells were washed with PBS and the cell membrane dye, DiO (10 μM), was added to group 1 and co-incubated for 5 min at 37 °C. Then, cells were washed with PBS observed by confocal laser scanning microscope (CLSM; Zeiss LSM 710).

Antimicrobial Assay. For the light-induced toxicity experiment, 100 μL (10^8 CFU/mL) bacteria *E. coli*, were dispersed in the solutions containing TBP-2 (10μg/mL), and incubated at 30℃ with a shaking speed of 200 rpm for 10 min. Then, the treated bacteria were washed and diluted in PBS, from which 100 μL (10^4 CFU/mL) bacteria were sprayed onto a LB agar plate. Part of plates were then exposed to WL (0.1 W/cm^2) for 5min, and part of plates were put in dark. Later, all the LB agar plates with treated bacteria were cultured at 37 ° C for 12 h. The images of the plates taken by a digital camera were used for counting the number of colony-forming units (CFU). The survival rate of bacteria was determined by dividing the CFU of bacteria incubated with PSs by the CFU of the control group performed in the absence of PSs and light[1].

Intracellular reactive oxygen species (ROS) generation. For determination of ROS levels *via* fluorescent imaging, CT26 cells were incubated for 0.5 h with 4 different groups: (1) PBS; (2) white light laser (L, 0.1w/cm^2, 10min); (3) AE; (4) AE+L. The TBP-2 concentration was 10 μg/mL in group 3, and 4. Then, the fluorescent dye, DCFH-DA (10 μM), was added and

co-incubated for 20 min at 37 °C. Then, cells in group 2 and 4 were exposed to laser radiation for 10 min. ROS level was determined by confocal laser scanning microscope (CLSM; Zeiss LSM 710). The above experiments were subsequently performed under hypoxic conditions, and the fluorescence images were recorded and analyzed by confocal laser scanning microscope (CLSM; Zeiss LSM 710).

Singlet oxygen ($^1O_2$) generation detection in cancer cells: SOSG was used as an $^1O_2$ indicator[4]. The AE (10μg/mL TBP-2) and SOSG were co-incubated with CT26 cells for 10min under normoxia or hypoxia condition. Then, cells were exposed to white laser radiation for 10 min. ROS level was determined by confocal laser scanning microscope (CLSM; IX81, Olympus, Japan).

Hydroxyl radical (OH•) detection: For OH• evaluation, the experiment procedure was same as $^1O_2$ measurement except using the HPF as the OH• indicator[4].

In vitro anticancer effect of AE. The phototoxicity was measured by MTT assay. CT26 cells were seeded in 96-well plates at a density of 5×10^4 cells per well and incubated for 12 h. Afterwards, cells were incubated for 4 different groups: (1) PBS; (2) white light laser (L, 0.1w/cm^2, 10min); (3) AE; (4) AE+L. The TBP-2 concentration was 10 μg/mL in group 3, and 4. Then, cells in group 2 and 4 were exposed to laser radiation for 10 min. Then, cells were washed with PBS for three times. At the end of the incubation, 5 mg/mL MTT PBS solution was added, and the plate was incubated for another 4 h. Finally, the absorbance values of the cells were determined by using a microplate reader (Emax Precision, USA) at 570 nm. The background absorbance of the well plate was measured and subtracted. The cytotoxicity was calculated by dividing the optical density (OD) values of treated groups (T) by the OD values of the control (C) (T/C × 100%)[5]. We then used the same method to verify the *in vitro* phototoxicity of AE with different TBP-2 concentration (0, 5 and 20 μg/mL). The above experiments were subsequently performed under hypoxic conditions.

***In vivo* bio-distribution study.** All mice in this study (6 weeks of age,15-20 g, male) were purchased from GemPharmatech Co.,Ltd. Jiangsu, China. All animals were housed in a specific pathogen-free (SPF) laboratory in the Animal Center of China Pharmaceutical University at 22 ± 1 °C temperature and 40-50% humidity under a 12-h light/dark cycle with free access to water and standard laboratory chow. All procedures were approved by the Institutional Ethics Committee for Animal Experimentation and were conducted in accordance with the 2nd Clinical Medical College (Shenzhen People's Hospital) of Jinan University. The CT26 orthotopic tumor model was used. When tumors reached a diameter of up to 7 mm (Twice the diameter of the intestine and monitored by the endoscope), tumor

bearing mice (n = 3) received an intravenous (i.v.) or intragastric injection of 100 μL PBS containing AP or AE (with a TBP-2 dose of 3 mg/kg). Mice were sacrificed at 24 h after injection to collect the tumors and major organs for imaging analysis and fluorescence intensity measurement by using the IVIS system. Capture condition: λex = 488 nm, λem = 600–700 nm. Because the intestinal tract has a strong background fluorescence in this band, the tumors were isolated for in vivo imaging. Ex vivo images at 6h and 12h post-AE injection were also conducted.

***In vivo* antitumor study.** The CT26 orthotopic tumor model was used. When tumors reached a diameter of up to 3 mm (monitored by the endoscope), tumor bearing mice were divided randomly into 4 groups (each group included 5 mice): (1) PBS+ interventional white laser (L); (2) AE; (3) AE+ external white laser irradiation (EI); (4) AE+L. The TBP-2 dose was 5 mg/kg in group 2, 3 and 4. The PDT (0.1 W/cm^2, 20min) was performed 24 h after intravenous injection. The treatment was conducted every 4 days for 14 days. Mice body weight was monitored every 2 days. After 14 days treatment, all the mice were sacrificed. The blood samples from these mice (≈1 mL) were collected for blood biochemistry analysis. Five main organs (heart, liver, spleen, lung and kidney) and tumors of all mice were harvested, washed with PBS, and fixed with paraformaldehyde for histology analysis. And the tumor tissues were imaged and weighed, and fixed in 4% neutral buffered formalin, processed routinely into paraffin, and sectioned at 4 μm. Then the sections were stained with Terminal deoxynucleotidyl transferase-mediated deoxyuridinetriphosphate nick end labeling (Tunel) and hematoxylin and eosin (H&E) and finally examined by using an optical microscope (BX51, Olympus, Japan).

Statistical analysis. Data analyses were conducted using the GraphPad Prism 5.0 software. Significance between every two groups was calculated by the Student’s t-test. *P < 0.01, **P < 0.005, ***P < 0.001.

Supplementary figures

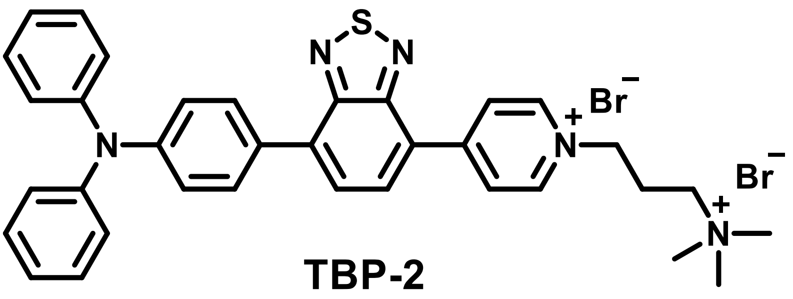

Figure S1. The structure of TBP-2.

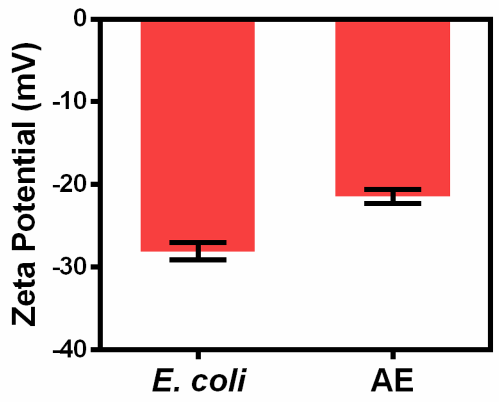

Figure S2. Zeta potential of *E. coli* and AE.

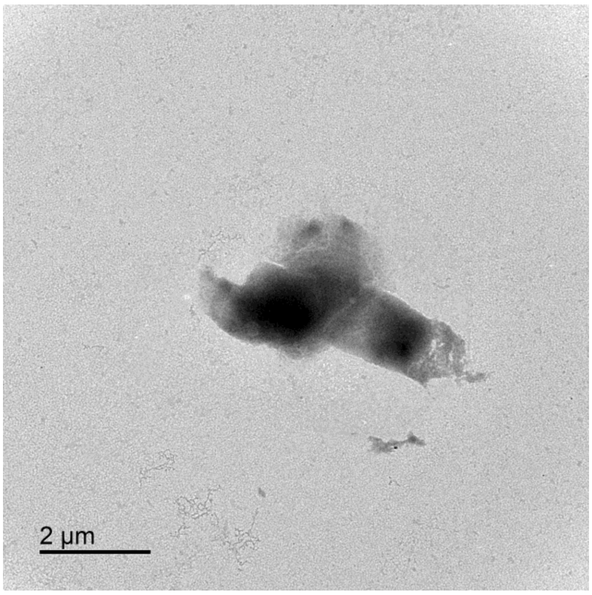

Figure S3. TEM image of AE after light irritation.

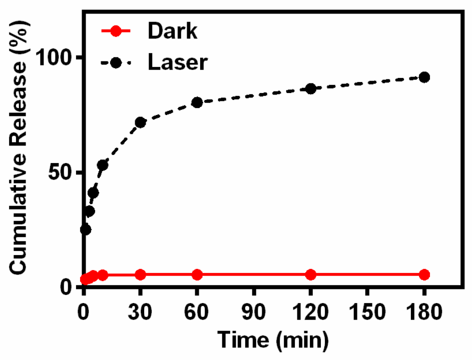

Figure S4. In vitro TBP-2 release profile at 37℃ from AE with or without laser irradiation.

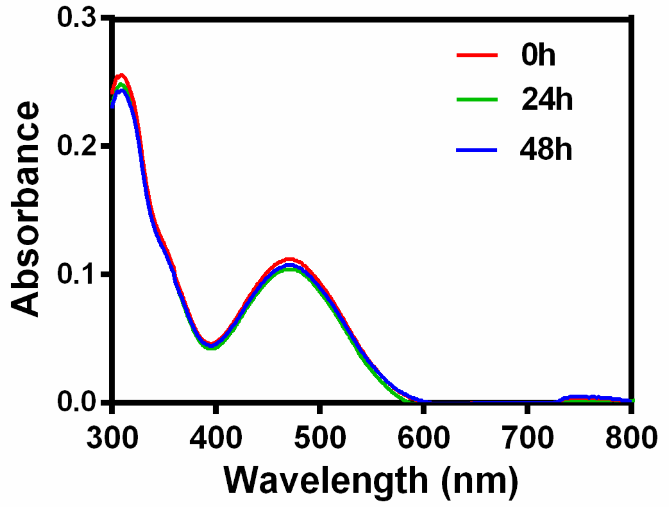

Figure S5. UV-vis spectra of AE in the process of culture.

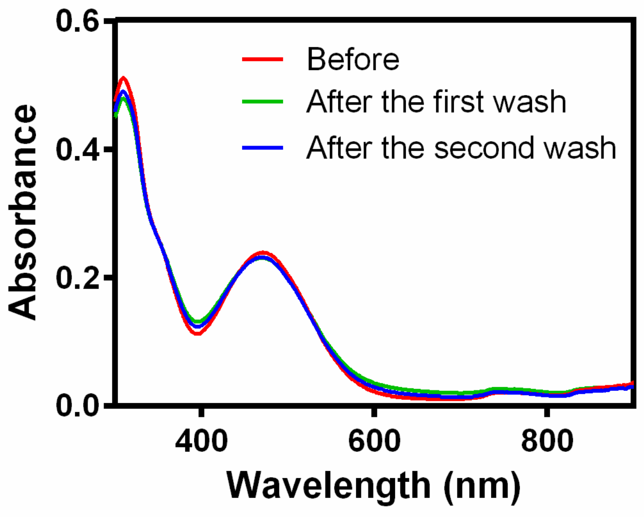

Figure S6. UV-vis spectra of AE before and after washing by PBS.

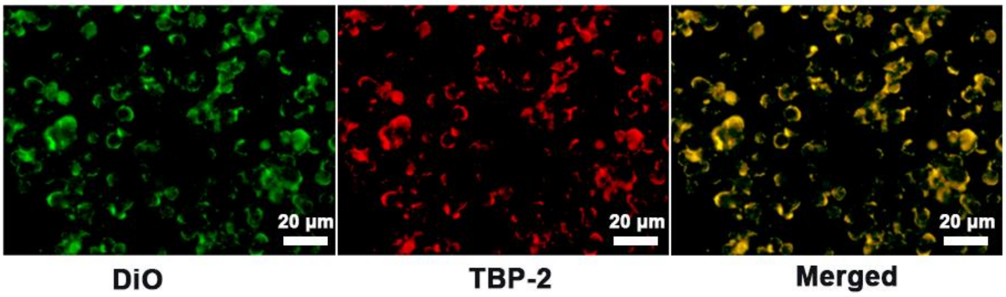

Figure S7. CLSM images of TBP-2 and DiO co-stained cancer cells.

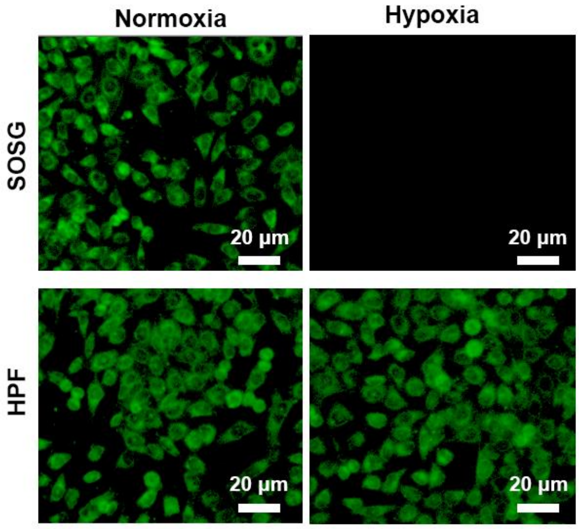

Figure S8. CLSM images of ROS in AE-treated CT26 cells in normoxia and hypoxia environments under white liaght laser irradiation using SOSG, and HPF as $^1O_2$, and •OH detection probe.

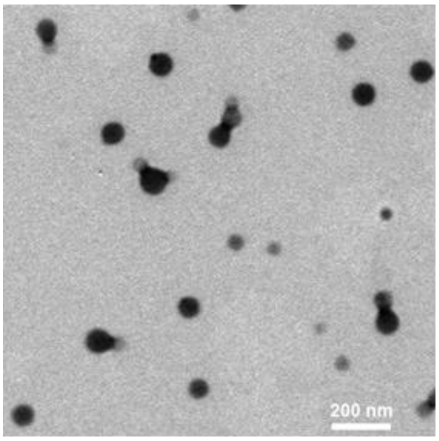

Figure S9. TEM image of AP.

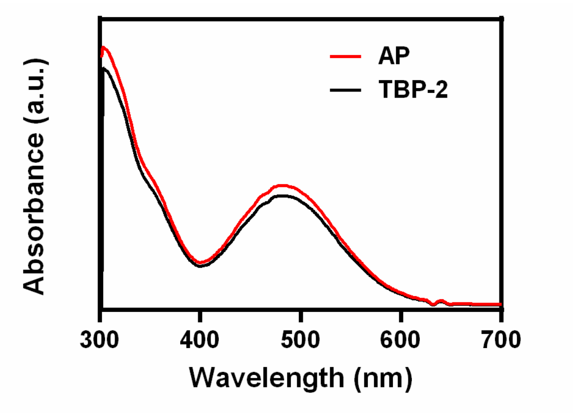

Figure S10. UV-vis spectra of AP and TBP-2.

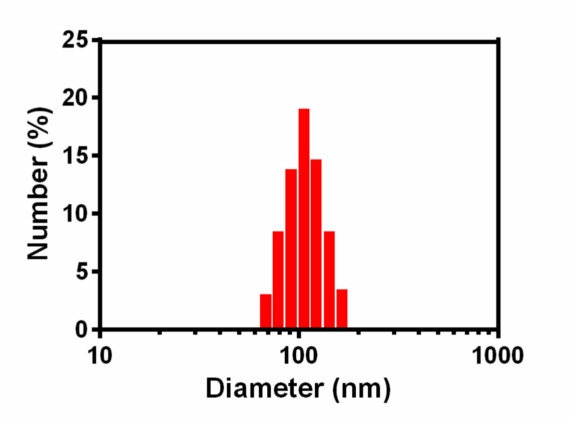

Figure S11. Hydrodynamic diameters of AP measured by DLS.

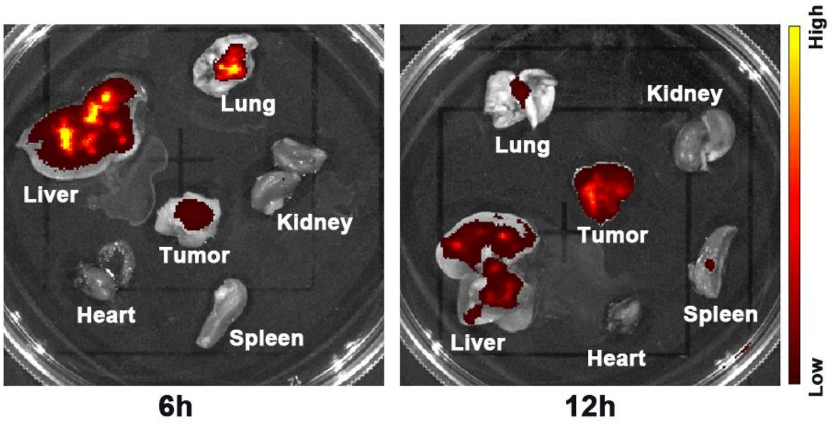

Figure S12. Ex vivo images in collected tumor tissues and organs in mice bearing orthotopic CT26 tumors at 6h and 12h post-AE injection.

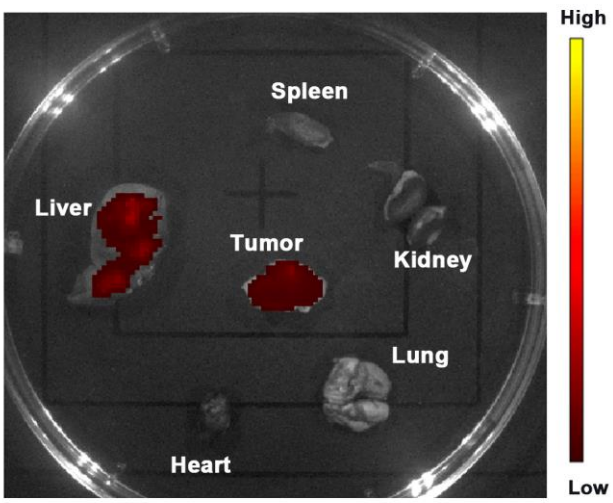

Figure S13. Ex vivo images in collected tumor tissues and organs in mice bearing orthotopic CT26 tumors 24 hours after oral administration of AE.

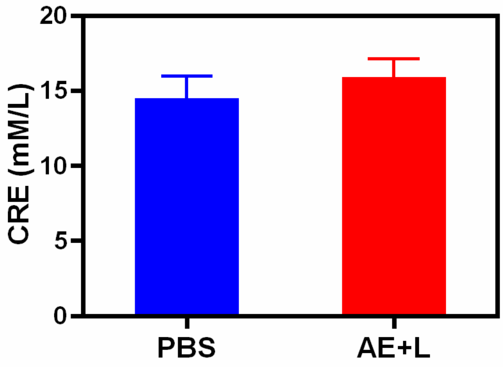

Figure S14. Blood biochemistry data of kidney function markers: CRE.

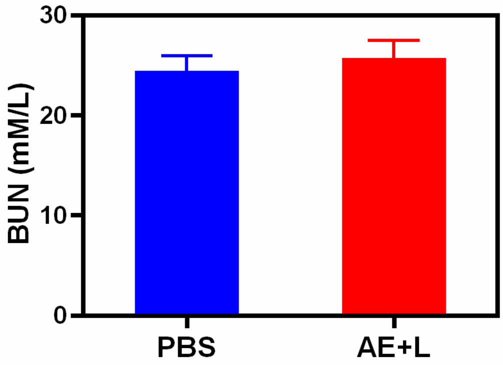

Figure S15. Blood biochemistry data of kidney function markers: BUN.

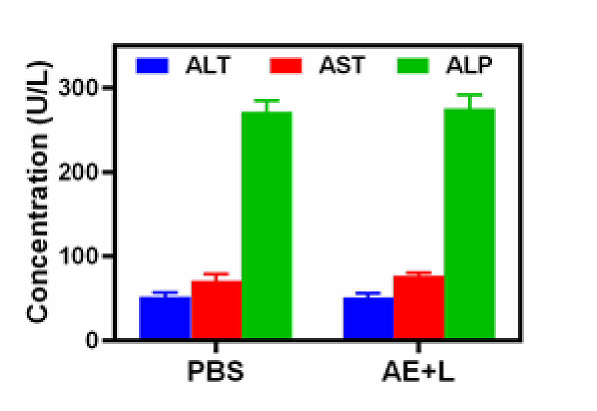

Figure S16. Blood biochemistry data of liver function markers: ALT, ALP, and AST.

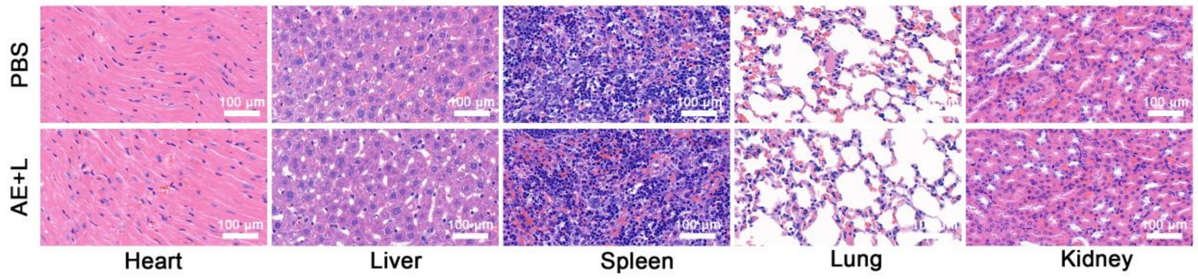

Figure S17. Histopathologic examination of the tissues including heart, liver, spleen, lung, and kidney from tumor-bearing mice after PBS or AE+L treatment.

References

[1] X. Shi, S. H. P. Sung, J. H. C. Chau, Y. Li, Z. Liu, R. T. K. Kwok, J. Liu, P. Xiao, J. Zhang, B. Liu, J. W. Y. Lam, B. Z. Tang, *Small Methods* **2020**, 4, 2000046.

[2] D. Zhu, Y. Duo, S. Meng, Y. Zhao, L. Xia, Z. Zheng, Y. Li, B. Z. Tang, *Angew. Chem., Int. Ed.* **2020**, 59, 2-10.

[3] X. Pan, L. Bai, H. Wang, Q. Wu, H. Wang, S. Liu, B. Xu, X. Shi, H. Liu, *Adv. Mater.* **2018**, 30, e1800180.

[4] K. Zhang, Z. Yu, X. Meng, W. Zhao, Z. Shi, Z. Yang, H. Dong, X. Zhang, *Adv. Sci.* **2019**, 6, 1900530.

[5] D. Zhu, M. Lyu, Q. Huang, M. Suo, Y. Liu, W. Jiang, Y. Duo, K. Fan, *ACS. Appl. Mater. Interfaces.* **2020**, 12, 36928.
